# Multiple Papillomes of the Larynx

**Published:** 1894-10

**Authors:** A. Rosenberg

**Affiliations:** First Assistant of the Polyclinic of the University for Throat and Nose, Berlin, N. W.; Mittelstrasse 55


### MULTIPLE PAPILLOMES OF THE LARYNX.

*Editor Buffalo Medical and Surgical Journal:*

Being employed in preparing a paper on the treatment of multiple papillomes of the larynx in children, I observe that the opinions regarding the methods usually adopted are very much at variance. Certainly one must differentiate here as elsewhere; neither can we, or dare we, treat all cases in the same manner. There is no unanimity of opinion as to whether it is better to operate or to treat the cases otherwise.

It would be of great value to obtain the opinion of the majority of laryngologists on this subject; hence, for the purpose of collecting statistics, I beg all who have experience to furnish me with full details of their cases as follows:

1. Age and sex of child; occupation of father.
2. Symptoms, (hoarseness, dyspnea, etc.,) laryngoscopical image.
3. Manner and date of operation.
4. Result of operation as regards voice and breathing. Death—cause? Relapse—when? Recovery—date of the last examination.
5. Have these cases already been published? If so, when and where?

All gentlemen who will aid me in this matter have my best thanks in advance for their kind coöperation.

DR. A. ROSENBERG,

First Assistant of the Polyclinic of the University for Throat and Nose, Berlin, N. W.

MITTELSTRASSE 55, September 10, 1894.

